# Landmark-based evaluation of a deformable motion correction for DCE-MRI of the liver

**DOI:** 10.1007/s11548-018-1710-1

**Published:** 2018-02-23

**Authors:** Jan Strehlow, Nadine Spahr, Jan Rühaak, Hendrik Laue, Nasreddin Abolmaali, Tobias Preusser, Andrea Schenk

**Affiliations:** 1 Fraunhofer MEVIS, Am Fallturm 1, 28359 Bremen, Germany; 2 Fraunhofer MEVIS Lübeck, Maria-Goeppert-Str. 3, 23562 Lübeck, Germany; 3 Städtisches Klinikum Dresden, Friedrichstr. 41, 01067 Dresden, Germany; 4 Jacobs University Bremen, Campus Ring 1, 28759 Bremen, Germany

**Keywords:** Motion correction, Liver, MRI, Image registration, Evaluation

## Abstract

**Purpose:**

Annotation of meaningful landmark ground truth on DCE-MRI is difficult and laborious. Motion correction methods applied to DCE-MRI of the liver are thus mostly evaluated using qualitative or indirect measures. We propose a novel landmark annotation scheme that facilitates the generation of landmark ground truth on larger clinical datasets.

**Methods:**

In our annotation scheme, landmarks are equally distributed over all time points of all available dataset cases and annotated by multiple observers on a per-pair basis. The scheme is used to annotate 26 DCE-MRI of the liver. A subset of the ground truth is used to optimize parameters of a deformable motion correction. Several variants of the motion correction are evaluated on the remaining cases with respect to distances of corresponding landmarks after registration, deformation field properties, and qualitative measures.

**Results:**

A landmark ground truth on 26 cases could be generated in under 12 h per observer with a mean inter-observer distance below the mean voxel diagonal. Furthermore, the landmarks are spatially well distributed within the liver. Parameter optimization significantly improves the performance of the motion correction, and landmark distance after registration is 2 mm. Qualitative evaluation of the motion correction reflects the quantitative results.

**Conclusions:**

The annotation scheme makes a landmark-based evaluation of motion corrections for hepatic DCE-MRI practically feasible for larger clinical datasets. The comparably large number of cases enables both optimization and evaluation of motion correction methods.

**Electronic supplementary material:**

The online version of this article (10.1007/s11548-018-1710-1) contains supplementary material, which is available to authorized users.

## Background

In dynamic contrast-enhanced magnetic resonance imaging (DCE-MRI), a contrast agent (CA) bolus is monitored over a series of MR images. By acquisition of temporally scheduled volume scans, different vascular structures and tissue characteristics are imaged. DCE-MRI of the liver is used for surgery planning, tumor diagnostics, and functional analysis [10, 17]. In clinical routine, a series of abdominal DCE-MRI typically includes 4–5 volumes: (*native*), *arterial*, *portal-venous*, *equilibrium*, and *late-venous* phase. In special cases, subsequent images are acquired for the analysis of liver function parameters. Typically, the volumes of the series are acquired under separate breath-holds and the respiratory state may differ slightly from volume to volume. The resulting motion between the scans needs to be corrected in order to fuse the information of different phases and to analyze contrast dynamics on a per-voxel basis. DCE-MRI motion correction is typically implemented by image registration methods. The aim of medical image registration is to find a transformation between two or more images that optimally aligns anatomically corresponding positions. Comprehensive reviews of image registration methods can be found in [15]. The assessment of registration quality of a given method is not trivial. Especially in deformable image registration (DIR), it can be misleading to judge quality based on the aligned images after registration [13]. The first time points of a hepatic DCE-MRI are particularly challenging for image registration: The presence of a contrast agent highlights different anatomical structures, leading to the highest temporal variation of image contrast within the series. Vascular features of the arterial phase, for instance, may not be visible in other phases at all.

### Related work

Several image registration-based motion correction methods have been proposed for DCE-MRI series of the liver. In an early publication, Mainardi et al. corrected for organ motion of two subsequent phases by a rigid alignment of the liver followed by a slice-wise DIR [9]. Registration quality was assessed based on similarity after registration, leaving unclear whether the deformable transformation was physically plausible. Tokuda et al. used a different method to evaluate registration quality of a motion correction for large 2D time series [16]. They calculated a marker for liver function on the series using a pharmacokinetic model. The model-fit error before and after registration was used to assess the registration quality. A DIR for 3D time series was presented by Hamy et al. [5]. This method employs a robust principal component analysis to separate image changes induced by motion from those induced by the contrast agent, limiting it to series with high and constant temporal resolution. Besides qualitative measures, Hamy et al. use synthesized data to quantitatively evaluate their method. Registered non-contrast-enhanced time series are manually segmented and contrast enhancements within the different tissue classes are simulated based on pharmacokinetic modeling to generate a pseudo-ground truth. A DIR that is suitable for aligning both common clinical hepatic DCE-MRI as well as longer time series was proposed by Papiez et al. [12]. The method was evaluated based on manually annotated landmarks and an analysis of the deformation fields. For liver-applications, however, the method was only evaluated on two cases. Recently, Feng et al. proposed a manifold-based registration framework for liver DCE-MRI, that also relies on a decomposition of motion- and CA-related image changes [2]. Motion correction quality was assessed via time-intensity curves computed from manually annotated regions as well as by several visual and thus qualitative indicators.

Most publications assess performance via qualitative and indirect measures [6]. Indirect measures typically involve pharmacokinetic modeling and potentially introduce biases or are limited to certain scenarios. Motion corrections aiming at clinical applicability should further be evaluated on large datasets exhibiting realistic variety that may be problematic to synthesize.

A well-established direct method for registration evaluation is to sparsely generate transformation ground truth by annotating corresponding landmarks in the scans to be registered [1]. The distances between corresponding landmarks after registration, short *landmark distances* (LD), are then used to calculate a metric for registration quality. Castillo et al. formulated two requirements for a meaningful landmark-based evaluation of DIR methods: First, the landmarks should be spatially well distributed over the region of interest and second, the annotations should be numerous to ensure certain statistical criteria. They annotated numerous landmarks in some pairs and estimated the pooled standard deviation of landmark distance after registration ${\bar{s}}_{\texttt {DIR}}^{p}$. The number of annotations that are needed to ensure that a 95% confidence interval (CI) of size $\pm d$ mm around the mean LD is then given by

1 $$\begin{aligned} N_{\pm d }^{\texttt {DIR}}=\Bigg ( \frac{2{\bar{s}}_{\texttt {DIR}}^{p}}{d} \Bigg )^2 \end{aligned}$$

The method of Castillo et al. is mostly used for evaluation of DIR algorithms on single image pairs. On evaluation datasets with multiple pairs it is assumed that the LD after registration is predominantly a property of the registration method and less dependent on differing motion between the pairs.

A common scheme for landmark annotation on time series is to automatically find distinctive features on a *fixed image* and to annotate them in the *moving images* to be registered [11]. Changing visibility of image features renders this strategy infeasible for early phases of hepatic DCE-MRI. Annotating numerous landmarks on multiple time points of multiple time series and by multiple observers is practically not feasible and thus rarely reported, as also pointed out in [6].

**Fig. 1:**
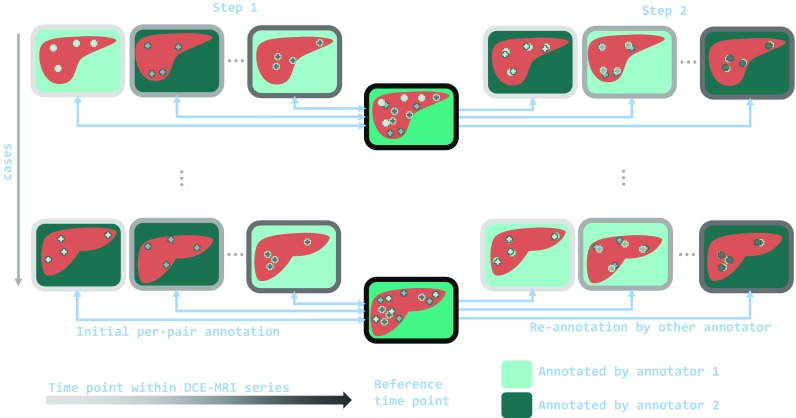
Schematic of the proposed annotation scheme for DCE-MRI datasets with multiple time point (horizontal direction) and multiple cases (vertical direction). One time point of the DCE-MRI series is selected as the fixed image. To account for varying feature-visibility, landmarks are annotated for pairs of fixed and moving image individually. In a first step, each annotator sets a small number of L landmarks on half of the pairs. Subsequently, the pairs are switched and the annotators re-annotate the other annotator’s fixed image landmarks

### Contribution 

Landmark-based evaluation of DCE-MRI motion corrections is hampered by the excessive workload of ground truth generation. We propose a new annotation scheme for DCE-MRI that makes landmark-based ground truth generation practically feasible for datasets with a large number of cases. We apply the scheme to collect ground truth data from 26 DCE-MRI series. Further, we combine a state-of-the-art DIR, a linear preregistration, and a novel liver segmentation to several exemplary motion correction methods for hepatic DCE-MRI and evaluate them using the generated ground truth. The high number of annotated cases enables us to split the ground truth into a training and an evaluation dataset. We show how the training dataset can be used to optimize the parameters of the motion compensation.

## Methods

### Ground truth

#### Annotation scheme

Landmark-based ground truth generation for DCE-MRI has two main challenges:

1. Changing visibility of image features within a DCE-MRI series makes it hard to annotate one anatomical position in all time points.
2. Annotation of a large number of landmarks in every time point of a DCE-MRI dataset with multiple cases is practically not feasible.

We propose an annotation scheme for DCE-MRI that mitigates those difficulties: First, independent landmarks are defined for each pair of fixed and moving image of the DCE-MRI series individually to promote the selection of landmarks that are assessable in both images of the pair. Second, we relax the requirement of numerous landmarks per scan pair but distribute numerous annotations equally over the whole dataset. An illustration of the annotation scheme is given in Fig. 1. In a first step, all pairs of fixed and moving images are divided into two halves and each half is assigned to one radiographer to annotate L corresponding landmarks per pair. In a second step, the pair sets are switched and the radiographers annotate the given fixed image landmarks of the other annotator a second time.

The statistical considerations on how many landmarks and how many cases are necessary for a statistically meaningful evaluation dataset are given in Section “Choice of annotation scheme parameters.”

**Fig. 2:**
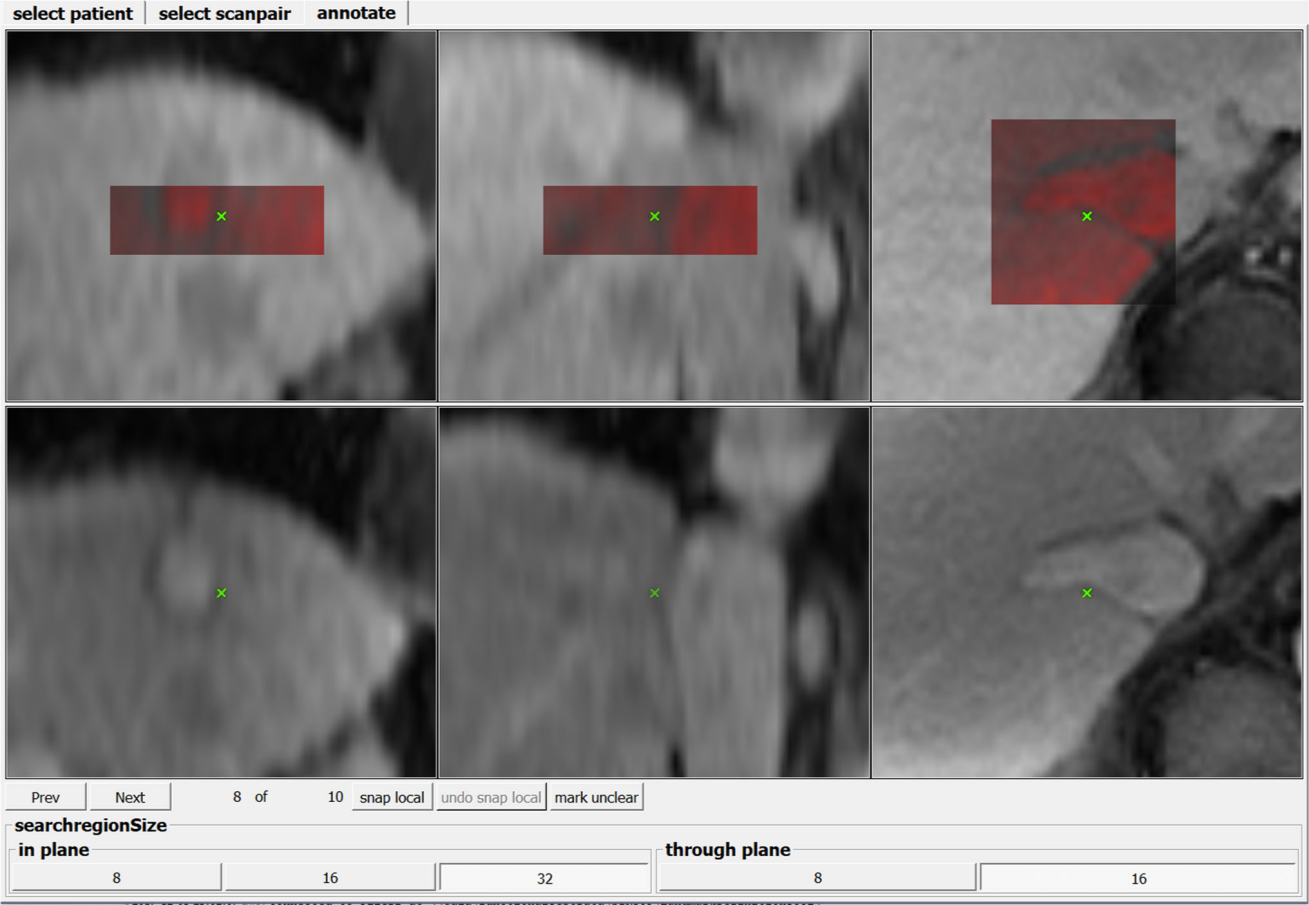
Screenshot of the in-house annotation tool used to define and find the landmarks. Fixed and moving images are displayed in separate rows and in three main directions. The user can switch between different zoom factors (zoom factor 4 shown). Once a landmark is defined in both images the user can overlay a local region around the moving image landmark in the fixed image (red overlay). The user can adjust the region size (bottom toolbar) as well as the windowing of the images and the overlay. The user can also perform a registration of the local region within the fixed image (button “snap local”)

#### DCE-MRI data

The annotation scheme is applied to DCE-MRI of 26 patients. The images were acquired on a 3T Discovery scanner (GE Healthcare Systems, USA) in an assessment step for a radioembolization intervention. Images were acquired in axial orientation with a standard LAVA sequence (flip angle: $12^{\circ }$). Eleven cases were acquired with a $256\times 256$ imaging matrix, 15 with a $512\times 512$ matrix, resulting in an in-plane voxel size between 0.74 and 1.72 mm (mean 1.11 mm). Slice thickness varied between 2 and 5 mm (mean 2.65 mm) and the mean voxel diagonal was 3.17 mm. Each DCE-MRI consists of five phases; the late-venous phase was acquired 15 min after CA (Primovist, (Bayer Healthcare, Germany) injection. In two cases the imaging matrix and field of view of the late-venous phase differed from the previous time points. For motion compensation, we reformatted these time points to achieve a consistent resolution within the series.

#### Annotation

Two trained radiographers were tasked to annotate the landmark ground truth. Our motion correction method registers the first 4 time points to the late-venous phase, resulting in four pairs of fixed and moving image per case and a total with a total of 104 pairs.

In principle, any annotation software could be used for landmarks annotation according to our scheme. We used an in-house annotation software (see Fig. 2), based on methods proposed by Castillo et al. [1]. The software simultaneously displays both volumes in axial, sagittal, and coronal orientation. To aid precise annotation, three tools are provided to the annotator:

- Display zoom factor can be switched between 1, 4, and 8.
- A small box around an annotated moving image landmark can be overlaid to the fixed scan.
- Moving image landmarks can be updated by a translational, normalized cross-correlation-based registration of the small box within the fixed image.

The box size can be varied to 8, 16, or 32 voxels in-plane, and 8 or 16 voxels through-plane. The annotators were encouraged to use different zoom levels and box sizes during annotation and were asked to choose landmarks that are distinct and spatially well distributed throughout the liver. During re-annotation, annotators could flag landmarks to indicate they were uncertain about the landmark position.

**Fig. 3:**
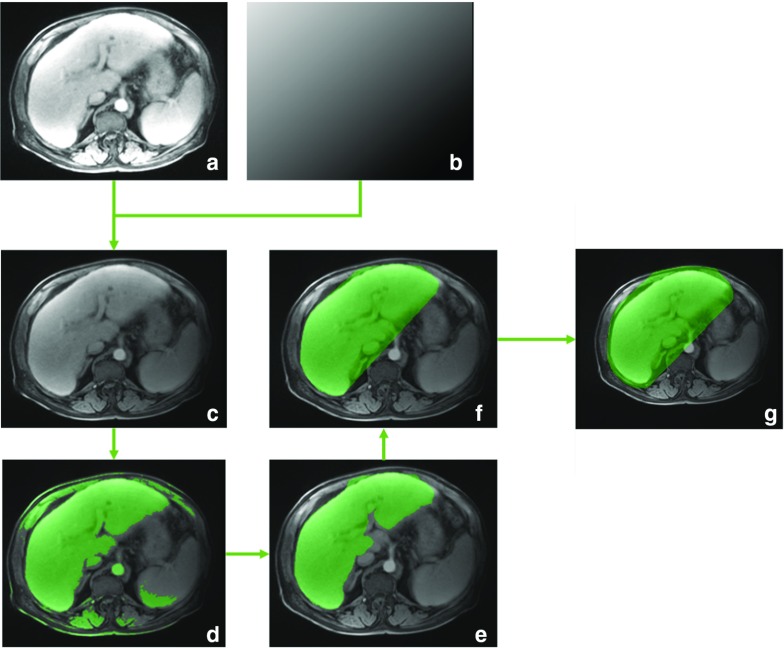
Steps of the liver segmentation. A mean intensity projection of all time points (**a**) is multiplied by a linear ramp (**b**) to give an image with high values within the liver region (**c**). On a threshold of this image (**d**) we subsequently compute a morphological opening (**e**) and the convex hull (**f**). The resulting mask is dilated again to include surrounding anatomy (**g**)

### Motion correction

Our method uses a sequence of pairwise registrations to correct for motion that occurs between the acquisition of the DCE-MRI volumes. We use the late-venous phase as the fixed image and employ a two-step pipeline: First, coarse differences in respiratory state are reduced by a rigid preregistration. Subsequently, organ deformations are corrected by a deformable registration. The preregistration step is restricted to the liver region by a coarse automatic liver segmentation that will be detailed below.

For deformable registration, we use a fast nonparametric method proposed by König et al. [8]. This method uses the similarity measure normalized gradient field (NGF) [4], which was successfully used for DCE-MRI of the kidney [6]. NGF has a parameter $\eta$, that is used to control the influence of features with different gradient magnitude. A high $\eta$ value will tend to diminish the influence of features with low gradients. Curvature regularization [3] is used as a regularization term. The influence of the regularization term is weighted by a factor $\alpha$.

For rigid preregistration, we use a method of Rühaak et al. [14] that also uses NGF as a similarity measure. To focus the preregistration to the liver, we restrict the evaluation of the similarity measure to an automatically computed coarse liver segmentation.

Our liver segmentation is based on the accumulation of CA in the liver (see Fig. 3). A mean intensity projection over all time points is computed giving an image with relatively high intensities in the liver and spleen regions (a). The liver region is emphasized over the spleen region by multiplying a linear ramp image to the projection that has its maximum value in the most anterior right position and its lowest value in the posterior left (b, c). After that, a region including the liver is extracted by applying a threshold at the $90\%$ quantile of image values (d). Non-liver parts in the resulting mask are reduced by a morphological opening with a large kernel of 10 mm (e). The convex hull of the resulting mask gives the coarse liver segmentation (f). To include anatomies around the liver, the segmentation is again dilated with a kernel size of 5 mm (g).

We will denote the algorithm including all steps the *full method* and two variants, without masking the preregistration (RMwom), and without preregistration (RMwop) the *reduced methods*.

#### Parameter optimization

Motion correction quality of our method is governed by the choice of regularization factor $\alpha$ and the edge parameter $\eta$. While algorithm parameters are often chosen empirically, a quantitative ground truth on numerous cases, as we built it on our data, enables a different strategy: We define six randomly selected cases as training data and excluded them from evaluation. The algorithm parameters $\alpha$ and $\eta$ are then optimized with respect to the mean LD (see Section “Evaluation”) on the training set. To this end, we calculate motion corrections for parameter combinations within a broad search range ($\eta =\{0.25, 1, 4, 16, 64\}$, $\alpha =\{ 4, 16, 64, 256, 1024\}$). Next, a focused search range ($\eta =\{0.5 , 0.71, 1, 1.41, 2 , 2.83\}$ and $\alpha =\{16, 22.63, 32, 45.26, 64, 90.51, 128\}$) is evaluated around the best performing parameters within the broad range. The best performing parameters of the second range are then used to evaluate our method on the remaining data. We compare the full method with optimal parameters (FMopt) to an empirically motivated parametrization of the method (FMemp) we used before the ground truth was available ($\eta =1,\alpha =100$).

### Evaluation

While landmarks are used to sparsely sample the transformation ground truth, they cannot be used to evaluate the physical plausibility of a registration. We thus augment the landmark-based evaluation by a check for foldings in the deformation field. Further, we evaluate the motion compensations over a qualitative measure.

#### Quantitative measures

We evaluate our method based on the LD and the absence of foldings in the deformation field.

The landmarks were annotated by two observers to compensate for possible annotation errors. Landmark annotation error can either stem from inaccuracies in the annotation of the same anatomical structure (*type A error*) or even inadvertent annotation of different anatomical structures of similar appearance (*type B error*). To mitigate erroneous evaluation results due to type B annotation errors, we calculate the LD with respect to the closer of the two landmarks. We calculate mean, median, minimum, as well as the 75, 90, 99, and 100% quantiles of the LD and compare the LD distributions after all motion correction variants with the Wilcoxon signed-rank test with a significance threshold of 0.05.

To check for the absence of foldings, each voxel of a deformation field is split into eight equally sized tetrahedrons. A folding is detected if a tetrahedron changes its orientation after transforming its corners with the deformation.

#### Qualitative measures

A motion correction should transform anatomical positions to consistent image positions over time. Time cut images (TCI) are a visualization technique that can be used to assess whether a motion correction achieves this. In a TCI, the image values on a one-voxel line through the volume at subsequent time points are stacked horizontally to yield a 2D image, i.e., the image displays the temporal evolution of images values on the line. Motion between the time points of the series will result in discontinuous horizontal lines. If the motion is compensated well, the horizontal lines appear straight (see green and gray arrows in Fig. 5b, c). We calculate TCI for three anterior–posterior lines on the slice with maximal liver extent. Within this slice, the lines were manually placed left, right, and in the center of the liver (see Fig. 5a). Pairs of TCI before and after motion compensation with FMopt are visually rated by an expert whether the temporal consistency of images features *improved*, *decreased* or is left *indifferent* by motion correction. TCI after all other methods are compared to the TCI after FMopt and rated whether the method performed *better*, *indifferent*, or *worse*.

A video showing all slices of a representative case before and after motion correction with FMopt is provided in the supplementary material.

### Choice of annotation scheme parameters

Our landmark annotation scheme has two parameters, the number of landmarks per pair *L* and the number of pairs *P*. The parameter *L* is chosen to fulfill the requirement that landmarks should be well distributed over the region of interest. We choose $L=10$ as a compromise between spatial coverage of the liver and induced workload.

**Table 1:** Statistics of the extent of the bounding box around all fixed image landmarks and average extents of the normal liver as reported in [7]

Direction	Mean (mm)	Min (mm)	Max (mm)	Normal liver extent from [7] (mm)
Left–right	154.6	56.8	263.4	200–255
Craniocaudal	116.9	29.7	200.8	150–175
Anterior–posterior	120.5	52.0	220.5	100–125

**Fig. 4:**
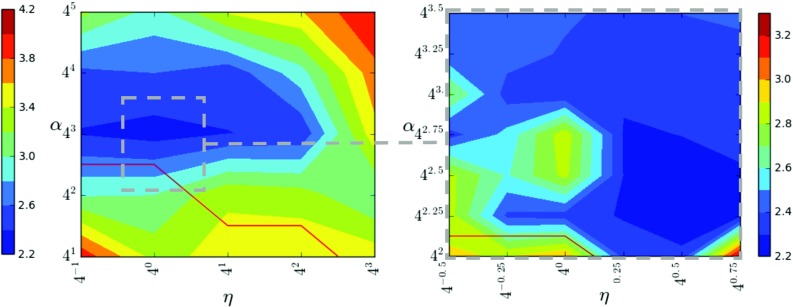
Median landmark distances (mm) for different parameter combinations in a broad (left) and a focused (right) search range. Foldings occurred in parameter combinations below the red line. Note that the contours are interpolated in between the sampled positions and that the scales of the two contour plots differ

For a coarse estimation of P we treat each pair as if it had only one annotation. Following the assumption that the LD after registration is mostly independent of the underlying motion we can then use Formula 1 to estimate the number of pairs that are necessary to ensure a certain size of a 95% CI. We aim on an evaluation method that has a 95% CI smaller than half the mean voxel diagonal and thus choose $\pm d = 0.7925$. Further, we estimate the standard deviation of the LD after registration with FMemp by annotating a single case with 50 landmarks per time point and computing the standard deviation after motion compensation ${\bar{s}}_{\texttt {FMemp}}=3.07$. Using Formula 1 we calculate $P>61$. To compensate for estimation errors we annotated 104 pairs. After annotation of the 104 pairs with 10 landmarks, we calculate the actual standard deviation of LD after the full method with empirical parameters to $s_\mathrm{FMemp}= 1.9$ mm. We estimate the number of pairs necessary for a 95% CI of size $2\times 0.7925$ as before and get $P>23$ as a lower bound. Consequently, we chose a training dataset that contains 24 pairs, i.e., six cases.

## Results

### Ground truth

In total, 990 landmarks were annotated on the 26 cases, each by two annotators. One hundred and two of those landmarks were marked as ‘uncertain’ and were thus excluded from the evaluation. Five time points exhibited strong intra-scan motion artifacts and could not be annotated at all. To give an estimate of the ground truth quality, we calculate the inter-observer distance (IOD), i.e., the distance between corresponding moving image landmarks of the two annotators. Mean IOD was 3.01 mm (median: 2.09 mm, min: 0 mm, max: 22.64 mm), which is smaller than the mean voxel diagonal. Overall annotation time per annotator for all landmarks was below 12 h, distributed over several sessions. Statistics for the spatial extent of fixed image landmarks as well as average normal liver extents reported in [7] are given in Table 1.

### Parameter optimization

Mean LD was computed for 65 different parameter combinations of $\alpha$ and $\eta$ on the training data. Figure 4 shows the mean LD as contour plots for the broad and the focused search range. Foldings were detected for several parameter configurations with low $\alpha$ and $\eta$ values (below red line). Within the focused search range, mean LD was minimal at a combination of $\alpha = 32$ and $\eta = 2$. Those parameters were used for FMopt.

**Table 2:** Statistics for LD after registration for our full method with optimal parameters (FMopt), the full method with empirically motivated parameters (FMemp), two reduced versions, one without masking the distance measure of preregistration (RMwom) to the coarse liver segmentation and one without preregistration (RMwop) and before registration

Method	Mean	Median	Min	q75%	q90%	q99%	Max	Foldings
FMopt	2.00	1.33	0.12	2.77	4.43	9.92	13.68	0
FMemp	2.10	1.45	0.08	2.76	4.57	9.31	17.49	0
RMwom	2.28	1.34	0.08	2.81	4.67	20.72	27.48	0
RMwop	2.24	1.37	0.06	2.84	4.73	14.36	24.73	0
Before reg.	10.23	8.42	1.10	14.22	23.82	28.66	31.91	–

All values are given in mm

### Registration quality

Table 2 lists LD statistics for our full method with optimal parameters (FMopt), the full method with empirically motivated parameters ($\eta = 1, \alpha =100$) (FMemp), the reduced versions without masking the preregistration (RMwom) and without preregistration (RMwop), as well as the landmark distances before registration. FMopt performs best on average as well as in the median and maximum LD. Both reduced methods only slightly reduce the maximum LD before registration and have elevated values in the 99% quantile. For the FMemp the median is slightly elevated ($+$ 9%) as compared to the full method. With respect to the Wilcoxon signed-rank test with a significance threshold of 0.05, the difference between FMopt and FMemp is statistically significant, the difference of FMopt to both reduced methods is statistically not significant.

**Fig. 5:**
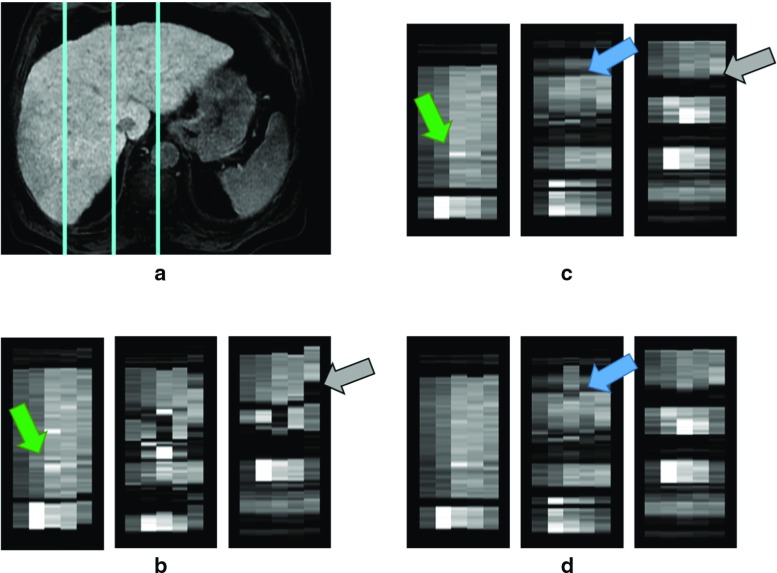
Time cut images (TCI) of three anterior–posterior lines of an exemplary case (**a**) before motion correction (**b**) after motion correction with the full method (**c**) and after motion correction without preregistration (**d**). The blue lines in (**a**) indicate the positions at which the TCI are calculated. The green and gray arrows point to structures that are temporally more consistent after motion compensation (green: blood vessel, gray: liver surface). The full method was rated as improving the temporal consistency in this case. The blue arrows in (**c**) and (**d**) mark a time point where the reduced method did not perform as well as the full method. The result of the reduced method was thus rated worse than the one of the full method for this case

Per evaluation case, three TCI pairs before and after motion compensation with FMopt were visually compared. In 51 TCI pairs, temporal consistency was rated as *improved*, in nine pairs it was rated *indifferent*. In all cases, at least one TCI pair indicated an improvement. The TCI of FMopt were compared to the TCI of the other methods on a per case basis. The TCI were mostly rated *indifferent*, only few cases were rated *worse* (FMemp:0, RMwom:4, RMwop:2) and none *better*. Time cuts for three lines of an exemplary case are displayed in Fig. 5. In this case, the result of FMopt was rated as *improved* as compared to the before motion compensation and RMwop was rated worse than FMopt because of a misalignment of a single time point (blue arrow). The green and gray arrows point to anatomical structures that show the improvement in temporal consistency.

The phase images of a representative case, before and after motion correction, are provided in a video as supplementary material. While continuously looping through the phases, the image slice is swept through the volume in the axial direction.

### Speed

Motion correction was performed on a desktop computer with standard hardware running Windows 10 (Intel Core I7 4770k, 32 GB RAM). Average computation time per scan pair was 62 and 246 s per case.

## Discussion

To enable landmark-based ground truth generation for DCE-MRI with multiple cases, we relax the requirement of numerous landmarks per scan pair but distribute numerous annotations equally over the whole dataset. In doing so, we accept that the statistical certainty of an evaluation measurement on a single scan pair is low. However, we compensate for this low per-pair certainty by drawing a high number of samples, i.e., we evaluate our metric on a high number of image pairs. This approach follows the assumption that the LD after registration is predominantly a property of the registration method and less dependent on the motion between time points of the DCE-MRI data. The qualitative analysis showed that the reduced methods did not follow this assumption in some cases: While both reduced methods produced very similar results to the full method in most cases, they failed to compensate motion in some time points. This behavior could be detected by elevated values in upper quantiles of the LD. The number of failing time points was, however, so low, that differences between FMopt and both reduced methods did not reach statistical significance, i.e., the null hypotheses that the LD after compared methods come from the same distribution cannot be discarded. FMemp does not show these outliers, and the comparison between FMemp and FMopt is statistically meaningful. In addition to common metrics such as the mean LD, evaluations based on our annotation scheme should thus also report quantiles of the LD. Since the number of landmarks per pair is comparably low, only statistics over numerous cases can be used for evaluation (see Section “Choice of annotation scheme parameters”). The comparison of TCI after different methods seems only suitable for the detection of large registration errors. Minuit difference in registration quality is hard to judge.

Having two annotators, our strategy to compensate for type B annotation errors is to calculate the LD with respect to the closer of the two annotated landmarks. Evaluation is thus “in doubt for the algorithm.” For an extended evaluation, the scheme can easily be generalized to an arbitrary number of annotators. For more that two annotators a majority vote could be used if the annotations disagree by a certain threshold.

Applying the proposed annotation scheme, we could annotate a comparably large dataset of 26 DCE-MRI in acceptable time with a plausible IOD. Manually annotated fixed image landmarks span a reasonable range when compared to mean extents of the normal liver. In certain time points, however, the landmarks are only distributed over a small area as indicated by relatively small values in the minimum extents in all three directions. At first glance, this may seem like an indication, that it is hard for an annotator to fulfill the demand of spatially well-distributed landmarks. Upon inspection of the cases with minimal spatial extent, the narrow spatial range of fixed image landmarks can be attributed to a heavy tumor load in the left lateral and left medial section (case with left-right minimum extent), an area of low contrast in the caudal part of the liver (craniocaudal minimum), and a partially resected liver (anterior–posterior minimum).

The quantitative measures in combination with the training dataset enabled a convenient search for physically plausible image registration with optimal parameters. During parameter optimization, detected foldings coincided with elevated mean LD values, as indicated in Fig. 4. Mean LD values within both search ranges show a substantial variation, and the difference between FMopt and FMemp is statistically significant. Optimizing parameters of a DCE-MRI motion correction with respect to a landmark ground truth can thus lead to an improvement of algorithm performance. With the best performing parameter set of the narrow search range, our motion correction method was able to achieve a mean LD of 2.0 mm.

The elevated maximum LD values and qualitative analysis of the two reduced methods indicate that the reduced methods do not achieve a good registration in a few time points. The case on which both reduced methods performed worst is a case with strong motion and the maximal distance of corresponding landmarks before registration of the entire dataset (31.9 mm). As compared to the reduced method, the proposed method with a masked preregistration is more robust with respect to a substantial variation of liver position within the series. The linear preregistration without restricting the distance measure to the liver region does not lead to an improvement.

## Conclusions

In this paper, we present a novel landmark annotation scheme that enables landmark-based evaluation of motion correction methods on large hepatic DCE-MRI datasets. With this scheme, 26 DCE-MRIs of the liver were annotated by two annotators within 12 h and with a mean inter-observer distance of 3.01 mm. With a high number of annotated cases, we used a part of the ground truth to optimize the parameters of a deformable DCE-MRI motion correction. Based on the ground truth, we compared several variants of our motion correction and found the ground truth to be sensitive to registration failures in single cases.

Parameter optimization led to a statistically significant improvement of the method. Reduced versions of our motion compensation without preregistration and without masking the preregistration failed to compensate the motion of single time points. Qualitative evaluation of the motion compensation on TCI was in-line with the findings of the quantitative landmark-based evaluation.

## Electronic supplementary material

Below is the link to the electronic supplementary material.

Supplementary material 1 (wmv 5867 KB)
